# Epidemiology of Mucormycosis in India

**DOI:** 10.3390/microorganisms9030523

**Published:** 2021-03-04

**Authors:** Hariprasath Prakash, Arunaloke Chakrabarti

**Affiliations:** 1 Medical Microbiology, Department of Public Health, International Higher School of Medicine, Issyk-Kul Regional Campus, Cholpon-Ata 722125, Kyrgyzstan; 2 Department of Medical Microbiology, Postgraduate Institute of Medical Education and Research, Chandigarh 160012, India; arunaloke@hotmail.com

**Keywords:** mucormycosis, incidence, diabetes mellitus, renal mucormycosis, *Rhizopus homothallicus*, amphotericin B

## Abstract

Mucormycosis is an angioinvasive disease caused by saprophytic fungi of the order Mucorales. The exact incidence of mucormycosis in India is unknown due to the lack of population-based studies. The estimated prevalence of mucormycosis is around 70 times higher in India than that in global data. Diabetes mellitus is the most common risk factor, followed by haematological malignancy and solid-organ transplant. Patients with postpulmonary tuberculosis and chronic kidney disease are at additional risk of developing mucormycosis in this country. Trauma is a risk factor for cutaneous mucormycosis. Isolated renal mucormycosis in an immunocompetent host is a unique entity in India. Though *Rhizopus arrhizus* is the most common etiological agent of mucormycosis in this country, infections due to *Rhizopus microsporus*, *Rhizopus homothallicus*, and *Apophysomyces variabilis* are rising. Occasionally, *Saksenaea erythrospora*, *Mucor irregularis*, and *Thamnostylum lucknowense* are isolated. Though awareness of the disease has increased among treating physicians, disease-associated morbidity and mortality are still high, as patients seek medical attention late in the disease process and given the low affordability for therapy. In conclusion, the rise in the number of cases, the emergence of new risk factors and causative agents, and the challenges in managing the disease are important concerns with mucormycosis in India.

## 1. Introduction

Mucormycosis is an angioinvasive disease that is characterised by tissue infarction and necrosis [1]. The clinical presentations of mucormycosis are classified on the basis of anatomic localisation, such as rhino-orbital-cerebral (ROCM), pulmonary, gastrointestinal, cutaneous, renal, and disseminated mucormycosis [2,3]. Patients with diabetes mellitus, haematological malignancy and chemotherapy, haematopoietic stem cells, and solid-organ transplant recipients on immunosuppressive therapy, with iron overload, on peritoneal dialysis, extensive skin injury, human immunodeficiency virus (HIV) infection, and voriconazole therapy are at increased risk of acquiring mucormycosis [2,3,4]. A considerable number of mucormycosis cases are reported in immunocompetent hosts [5,6,7]. Though mucormycosis is globally distributed, certain risk factors, clinical forms, and causative agents of the disease are prevalent in India.

Uncontrolled diabetes mellitus is the most common underlying disease associated with mucormycosis in India [5,6,8], in contrast to haematological-malignancy patients and solid-organ transplant recipients in developed countries [3,7,9]. Nevertheless, recent reports from India identified haematological malignancy and solid-organ transplant recipients as important risk factors, but the overwhelming number of patients with uncontrolled diabetes overshadows the picture [5]. The ROCM type is the most common form of the disease in India, followed by the pulmonary and cutaneous types [5,6]; however, the pulmonary form is the most common clinical presentation in developed countries [7]. The cutaneous type is commonly seen in patients with trauma or burns [5,6]. Isolated renal mucormycosis in a healthy host is a unique clinical presentation in India [10,11].

The pathogens associated with mucormycosis varies considerably between India and developed countries [12]. Globally, *Rhizopus arrhizus* is the commonest cause of mucormycosis [3,12]. The *Apophysomyces* species ranks second in India compared to the *Lichtheimia* species in developed countries [12]. Infections due to *Rhizopus microsporus* and *Rhizopus homothallicus* are rising in India [5,6,13]. In the present review, we discuss the epidemiology, risk factors and underlying diseases, causative agents, and clinical outcomes associated with mucormycosis in the Indian population.

## 2. Mucormycosis Prevalence and Incidence in India

The annual incidence of mucormycosis reported from different case series in India is shown in Table 1. Data from the three successive case series by Chakrabarti et al. from a single centre were pooled together in this review for a better extrapolation of the Indian picture [14,15,16]. Chakrabarti et al. showed an increasing trend of mucormycosis from a single centre at successive periods, with an annual incidence of 12.9 cases per year during 1990–1999 [14], 35.6 cases per year during 2000–2004 [15], and 50 cases per year during 2006–2007 [16]. The overall numbers increased from 25 cases per year (1990–2007) to 89 cases per year (2013–2015) [5]. The rise in incidence over the years at that centre may be due to improved awareness and expertise in diagnosing the disease, though the possibility of a real rise in incidence cannot be ruled out. A 10-year study from Southern India (Tamilnadu) showed an annual incidence of 18.4 cases per year during 2005–2015 [17]. Another study from Tamilnadu reported 9.5 cases per year during 2015–2019 [18]. A multicentre study across India reported 465 cases from 12 centres over 21 months; the study reported an annual incidence of 22 cases per year, and an average of 38.8 cases for each participating centre [6]. Though invasive aspergillosis is given importance among invasive mould infections in intensive-care units (ICUs), a multicentre study in Indian ICUs reported mucormycosis in a considerable (14%) number of patients [19]. Sindhu et al. reported mucormycosis at 12% in ICU patients at a single centre from North India [20]. Without population-based estimates, it is difficult to determine the exact incidence and prevalence of mucormycosis in the Indian population. The computational-model-based method estimated a prevalence of 14 cases per 100,000 individuals in India [21]. The cumulative burden ranged between 137,807 and 208,177 cases, with a mean of 171,504 (SD: 12,365.6; 95% CI: 195,777–147,688) and mean attributable mortality at 65,500 (38.2%) deaths per year [12,21]. The data indicates that the estimated prevalence of mucormycosis in India is nearly 70 times higher than the global data, which were estimated to be at 0.02 to 9.5 cases (with a median of 0.2 cases) per 100,000 persons [12].

## 3. Underlying Disease and Risk Factors

Table 1 shows the risk factors and underlying diseases associated with mucormycosis in India. Diabetes mellitus is the most common underlying disease, followed by haematological malignancies and solid-organ transplants. However, mucormycosis in the immunocompetent host is an alarming threat in the Indian population [5,6,8,17].

Diabetes mellitus was reported in 54–76% of cases (Table 1). Of those patients, 8–22% had diabetic ketoacidosis. The prevalence of mucormycosis was reported at 0.16–1.72% in patients with diabetes mellitus from North India [23,24]. Prakash et al. reported a higher prevalence of diabetes mellitus as a risk factor in North India (67%) compared to South India (22%) [5]. However, no such regional variation was noted in recent case series with regard to South India (65.2–76.3%) [17,18], North India (54–62.2%) [5,14,15,16,22], and Western India (55.6%) [8]. Similar to India, diabetes mellitus is a major risk factor in mucormycosis in Mexico (72%), Iran (75%), and the USA (52%) [12,25]. In comparison, the prevalence of diabetes in mucormycosis is lower (17–23%) in European countries [12,25].

Due to the lack of regular health check-ups in the Indian population, the diagnosis of mucormycosis unmasked diabetes in 43% of patients from North India [15], 40% in Western India [8], and 24% in South India [17]. These data signify the need for regular health check-ups in the Indian population. A recent estimate showed that 463 million adults (20–79 years), and 1 million children and adolescents under the age of 20 globally live with diabetes, which may rise to 578 million in 2030 [26]. China (116.4 million) and India (77 million) are at the top of the diabetes chart globally, followed by the USA (31 million). The situation is alarming in India, as the estimated diabetic population may rise to 101 million in 2030 [26]. Simultaneously, the expected rise of mucormycosis cases may worsen the condition.

Haematological malignancy (HM) is a risk factor in 1–9% of mucormycosis patients in India [25], compared to 38–62% in Europe and the United States [12]. Only a few studies documented mucormycosis prevalence in HMs from India [27,28]. A total of 781 acute leukaemia cases analysed from North India showed the prevalence of proven mucormycosis at 1.4% [27]. A study from South India on acute myeloid leukaemia patients reported the prevalence of proven mucormycosis cases at 0.9% [28].

Solid-organ transplantation (SOT) is a risk factor in 2.6–11% of mucormycosis cases from India (Table 1), compared to 7–14% from global data [2,3]. The prevalence of mucormycosis in renal-transplant recipients in India varies from 0.05% to 2.7% [29,30,31,32,33,34], compared to global data of 0.04–0.05% [35]. Multiple retrospective studies on renal-transplant recipients from South India documented mucormycosis prevalence at 0.56–1.52% [31,32]. A study from Western India (Gujarat) documented mucormycosis at 1.2% in renal-transplant recipients [30]. In North India, a group of authors conducted two retrospective studies on invasive fungal infections (IFIs) in renal-transplant recipients at different periods (1977–2000 and 2014–2017), and they documented the prevalence of mucormycosis at 2% and 2.7%, respectively [33,34]. These findings indicates that mucormycosis in renal-transplant recipients is more common in India than it is in developed countries.

In India, 3–26% of mucormycosis cases are recorded from the immunocompetent host (Table 1), compared to 18–19% globally [2,3]. Cases in India often present with cutaneous or isolated renal mucormycosis. Trauma is a risk factor in 7.5–22% of mucormycosis cases in India (Table 1). Majority of the patients present with cutaneous mucormycosis after trauma, burns, and nosocomial infections at the surgery or injection site. Chander et al. from North India reported cutaneous mucormycosis in patients with post-intramuscular injections in the gluteal region [22]. Another study from North India reported that 9% of the mucormycosis cases are nosocomial in origin [16]. Contaminated intramuscular injections and surgery, adhesive tapes, and endobronchial tubes were sources of infection in nosocomial mucormycosis [16,22,36,37]. Isolated renal mucormycosis in an immunocompetent host is an emerging entity in India; the pathogenesis of the disease is still not known [38,39].

Other predisposing factors associated with mucormycosis in India are chronic kidney disease (CKD), steroid therapy, pulmonary tuberculosis, and chronic obstructive pulmonary disease (COPD) [5,6]. CKD is a new risk factor for mucormycosis in India [5,15,40]. Studies from India reported that mucormycosis patients had CKD in 9–32% of cases [5,6,16]. Similarly, a study from Turkey reported that 18% of the patients with mucormycosis had chronic renal insufficiency [41]. Pulmonary tuberculosis and COPD were seen in 7–46% of patients with mucormycosis [6,19,20]. A few cases of breakthrough mucormycosis after voriconazole treatment were reported in India [42,43]. Other risk factors reported in India included intravenous drug use, autoimmune disease, HIV infection, immunosuppressant drugs, malnutrition, and ICU stay (Table 1).

## 4. Clinical Forms of Mucormycosis

**Figure 1:**
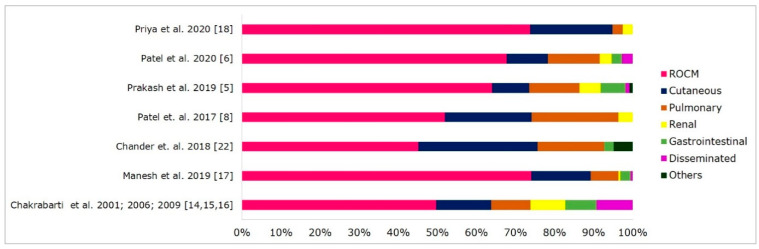
Clinical forms of mucormycosis in India. Abbreviations: ROCM, rhino-orbital-cerebral mucormycosis. Others included mucormycosis of the oral cavity, otitis media, subglottis, and bone infections.

**Figure 2:**
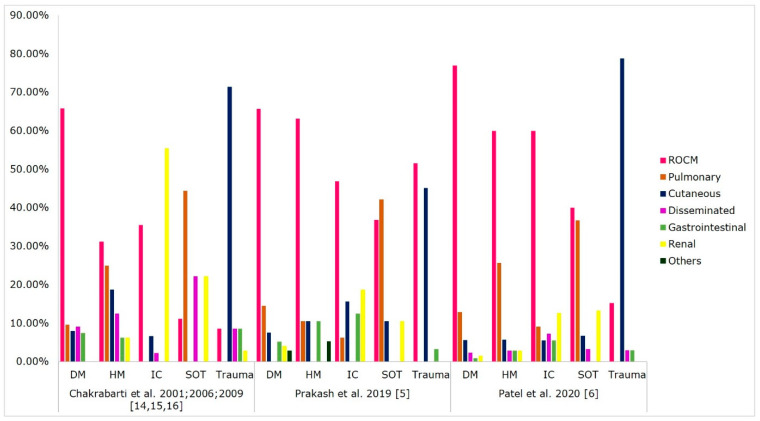
Risk factors associated with clinical forms of mucormycosis. Abbreviations: ROCM, rhino-orbital-cerebral mucormycosis; DM, diabetes mellitus; HM, haematological malignancy; IC, immunocompetent; SOT, solid-organ transplant.

On the basis of the anatomical site of involvement, the clinical form of mucormycosis reported in various case series from India is shown in Figure 1. ROCM mucormycosis is the commonest form (45–74%), followed by cutaneous (10–31%), pulmonary (3–22%), renal (0.5–9%), gastrointestinal (2–8%), and disseminated infections (0.5–9%). Other unusual sites of infection reported in the literature from India are breast [44], ear [5], spine [45,46], heart [47,48], and bone infections [49,50]. Figure 2 describes the underlying disease and risk factors associated with clinical forms of mucormycosis.

Diabetes mellitus is a common predisposing factor for the ROCM type of disease. A recent multicentre study from India reported that 77% of ROCM cases were in the diabetic population [6]. Different case series focussed on ROCM cases from India reported diabetes as a risk factor in 80–100% of cases [51,52,53,54,55,56]. Trauma is a risk factor for the ROCM type (15–52%), mainly after unhygienic dental procedures during tooth extraction [5,6,57,58].

Pulmonary mucormycosis is commonly associated with SOT recipients (37–44%), haematological malignancy (10–26%), and diabetes mellitus (10–14%) in Indian patients (Figure 2). These findings were similar to those of global data [2,3]. In Europe, haematological malignancy (34–44%) is the most common risk factor associated with pulmonary mucormycosis, followed by diabetes mellitus (13–14%) [7,59]. A review on pulmonary mucormycosis reported haematological malignancy (40%), diabetes mellitus (36%), CKD (17%), and SOT (6%) as significant underlying diseases [60]. In India, postpulmonary tuberculosis (38%) is a new risk factor for pulmonary mucormycosis [5].

The cutaneous type is seen in 10–31% of patients with mucormycosis after trauma following road traffic accidents, burn wounds, intramuscular injection, intravenous catheters, adhesive tapes, and surgical-site infections [5,22,61]. In India, 45–79% of cutaneous mucormycosis patients had trauma. Kaushik et al. reviewed cutaneous mucormycosis cases from India and reported trauma as a risk factor in 59% of the cases, followed by diabetes mellitus (28%) and malignancy (6%) [61]. A global study on cutaneous mucormycosis reported that 43–67% of patients were immunocompetent hosts, and other risk factors were diabetes mellitus (10–15%), malignancy (12–23%), and SOT (5–16%) [2,3,62].

Gastrointestinal mucormycosis accounts for 2–8% of cases from India (Figure 1). About 60% of the gastrointestinal cases are in paediatric patients, especially premature neonates (83%) [63]. Patra et al. reported gastrointestinal mucormycosis in 20% of neonates with suspected necrotising enterocolitis, and 83% of them were preterm neonates [64]. About 25–50% of patients with gastrointestinal mucormycosis had diabetes mellitus as a risk factor in India [5,6]. A review on gastrointestinal mucormycosis in immunocompetent hosts reported diabetes mellitus (24%) and peritoneal dialysis (16%) as significant risk factors in adults, and broad-spectrum antibiotic use (47%) and malnourishment (26%) in children [63]. Patients with SOT (52%) and haematological malignancy (35%) are also at risk of developing gastrointestinal mucormycosis [65].

Renal mucormycosis in an immunocompetent host is a unique clinical entity in India. Different case series from India reported that 33–100% of renal mucormycosis cases were in an immunocompetent host [12]. Prakash et al. reported haemodialysis and CKD as significant risk factors in renal mucormycosis patients [5]. Isolated renal mucormycosis can affect unilateral or bilateral kidneys [38,39]. Patients present with fever, flank pain, haematuria or dysuria, acute kidney injury, and white flakes in urine [38,39]. Computed tomography (CT) or ultrasound may help in achieving an early diagnosis of renal mucormycosis. Enlarged kidneys with or without hypodensities, perinephric stranding, and thickened Gerota’s fascia are classical imaging findings in these group of patients [38,39].

## 5. Causative Agents of Mucormycosis

**Figure 3:**
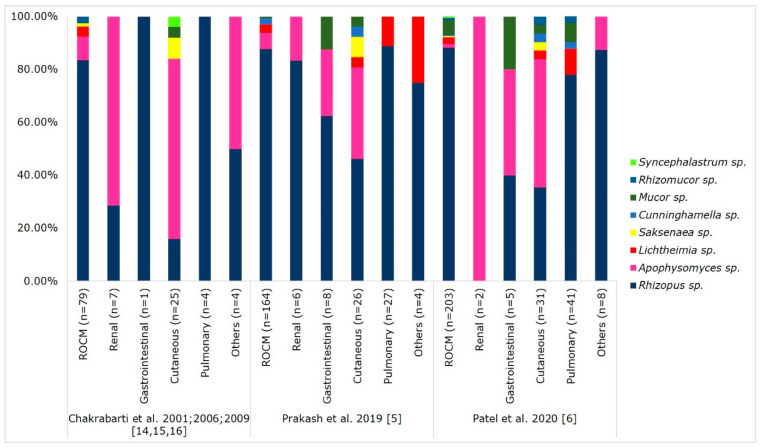
Mucorales spectrum associated with clinical forms of mucormycosis. Abbreviations: ROCM, rhino-orbital-cerebral mucormycosis. Others included mucormycosis of oral cavity, otitis media, subglottis, bones, and disseminated infections.

Mucorales are thermotolerant saprophytic fungi found in decaying organic matter and soil samples [66,67]. An ecological study on Mucorales in Indian soils documented the isolation of pathogenic species such as *Rhizopus*, *Lichtheimia*, *Cunninghamella*, *Rhizomucor*, and *Apophysomyces* [66]. Similarly, aeromycological analysis in a community and hospital setting from India reported the isolation of pathogenic Mucorales in air samples [67]. The taxonomy of Mucorales is evolving; a total of 11 genera and 27 species were described as causative agents of mucormycosis [12,68]. Table 2 and Figure 3 show the spectrum of isolated Mucorales and their association with clinical types in the Indian population.

*Rhizopus arrhizus* is the most common agent causing mucormycosis in India and globally. However, the spectrum of agents causing this disease in India is considerably large. Recent studies reported a rise in mucormycosis cases due to *Rhizopus microsporus* and *Rhizopus homothallicus* [5,6,13,69]. *Rhizopus* species are associated with ROCM mucormycosis [3,5,6], and this finding correlates well with the abundant presence of *Rhizopus* species in soil and air samples [66,67]. *Apophysomyces variabilis* is the second commonly isolated agent. India accounts for approximately 60% of reported cases in the literature due to *Apophysomyces* species [12,70], and the fungi cause cutaneous mucormycosis in the form of necrotising fasciitis [3,5,6,70]. The fungi were abundantly isolated from Indian alkaline soil with low nitrogen content [65]. Rarely, the agent can cause the ROCM and renal forms of mucormycosis [5,38,71]. An aeromycological survey showed the presence *of Apophysomyces* species in air samples, which may explain the source in ROCM mucormycosis [67]. A study from South India reported that 29% of cases due to *Apophysomyces* species were nosocomial in origin [70].

*Lichtheimia* species contribute 0.5% to 13% of cases from India. Chander et al. reported that most of the cases in India are due to *L. ramosa* [22]. Other Mucorales associated with mucormycosis in India are *Rhizomucor pusillus*, *Cunninghamella* species, *Mucor* species, *Syncephalastrum* species, and *Saksenaea* species (Table 2). Mucormycosis due to rare pathogens such as *Saksenaea erythrospora*, *Mucor irregularis*, and *Thamnostylum lucknowense* are also reported [22,72,73,74].

## 6. Treatment and Outcome of Mucormycosis

**Figure 4:**
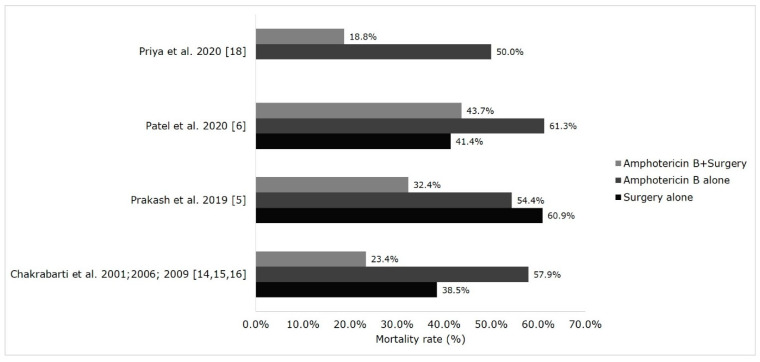
Modes of therapy and mortality rate in Indian population. The data shown in figure for the study Patel et al. 2020 [6] was extracted from the master sheet provided by the authors.

**Figure 5:**
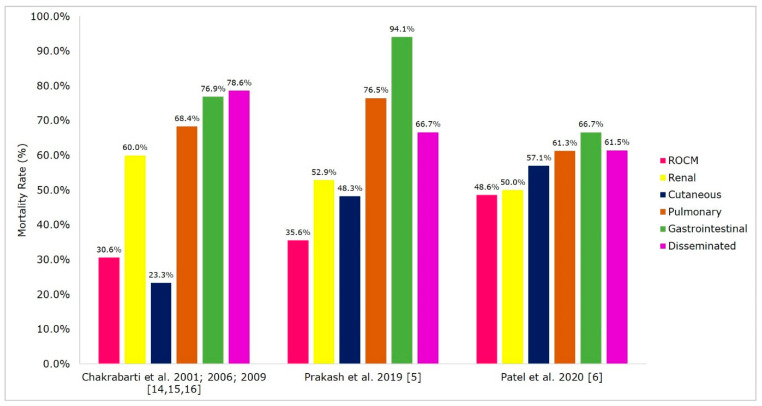
Morality rate in clinical forms of mucormycosis in India. Abbreviations: ROCM, rhino-orbital-cerebral mucormycosis.

The treatment and outcome of mucormycosis in Indian patients are depicted in Figure 4 and Figure 5. The treatment of mucormycosis involves the early initiation of therapy, the surgical debridement of infected tissue, antifungal therapy, and managing the underlying disease. Amphotericin B (AmB) is the first-line drug of choice; subsequently, posaconazole and isavuconazole are prescribed [75]. The major drawbacks in managing mucormycosis in India are a gap in treatment protocol and the financial constraints of patients that they cannot afford liposomal AmB [6,8]. Existing data showed that the mortality rate was low in patients treated with a combination of AmB and surgical debridement of the infected tissue (19–44%) compared with AmB monotherapy (50–61%) (Figure 4), these findings are in concordance with global data [76]. Posaconazole and isavuconazole were used as salvage therapy in the treatment of mucormycosis [75]. A study from South India assessed the safety and efficacy of posaconazole in ROCM patients. The study reported no mortality; 66.6% of patients had complete resolution of the disease, and the rest a significant reduction of the disease [77]. The new anti-Mucorales drug isavuconazole showed comparable efficacy to AmB [78], however, it is recently introduced in Indian market and its efficacy is still to be assessed in this country. The mortality rate of mucormycosis in India is in the range of 28–52% [5,6,14,15,16,17]. The mortality rate in different clinical forms of mucormycosis reported from India are ROCM (31–49%), pulmonary (61–77%), cutaneous (23–57%), gastrointestinal (67–94%), and disseminated (62–79%) (Figure 5); these findings are similar to those in global data [2,3].

## 7. Conclusions

The exact prevalence of mucormycosis in India is unknown, though the estimated prevalence is much higher than that in developed countries. The possible reason for the high prevalence is the abundant presence of Mucorales in the community and hospital environment, large number of susceptible hosts especially diabetics, and the neglect for regular health check-ups of Indian population. A considerable number of patients are ignorant of diabetes status till they acquire mucormycosis. Though uncontrolled diabetes is a common risk factor in all types of mucormycosis, it is significantly associated with ROCM type. Other emerging risk factors of mucormycosis are pulmonary tuberculosis, chronic kidney disease, and critically ill patients. Isolated renal mucormycosis in an immunocompetent host is a unique clinical entity and requires more studies on pathogenesis. Like in the global data, *Rhizopus arrhizus* is the most common causative agent isolated in all clinical forms of mucormycosis. However, the spectrum of agents causing the disease is considerably large in India. *Apophysomyces* and *Saksenaea* species are common agents causing cutaneous mucormycosis. Newer species like *Rhizopus homothallicus, Rhizopus microsporus, Mucor irregularis*, *Thamnostylum lucknowense*, and *Saksenaea erythrospora* are emerging in India and require expertise in laboratory identification. The broad spectrum of agents emphasises the need to improve routine clinical laboratory facilities to identify rare Mucorales associated with mucormycosis. Mortality associated with mucormycosis in India is considerably high due to delays in seeking medical attention and diagnosing the disease, and challenges in managing the advanced stage of infection. It is necessary to conduct population-based studies in India to determine the exact prevalence of mucormycosis in diverse at-risk populations, which would help draw stakeholder attention to the early diagnosis and managing the disease. Though AmB is routinely used in the treatment of mucormycosis, it is important to study the role of newer antifungal agents such as isavuconazole in the treatment of mucormycosis in the Indian population.

## Figures and Tables

**Table 1:** Annual incidence and risk factors of mucormycosis in India.

Parameters	Chakrabarti et al., 2001; 2006; 2009 [14,15,16]	Manesh et al., 2019 [17]	Chander et al., 2018 [22]	Patel et al., 2017 [8]	Prakash et al., 2019 [5]	Patel et al., 2020 [6]	Priya et al., 2020 [18]
Study centre	1	1	1	2	4	12	1
Study period	1990–2004; 2006–2007	2005–2015	2010–2014	January 2013– May 2015	2013–2015	January 2016–September 2017	2015–2019
Study duration	15 years 6 months	10 years	5 years	2 years 5 months	3 years	1 year and 9 months	4 years
Place of study	Chandigarh (North India)	Tamilnadu (South India)	Chandigarh (North India)	Gujarat (West of India)	North and South India	Across India	Tamilnadu (South India)
Total cases	382	184	82	27	388	465	38
Mean annual incidence	24.5	18.4	16.4	-	-	-	9.5
Male: female ratio	2.4:1	2.5:1	2.04:1	2.3:1	2.3:1	2.3:1	2.8:1
*Paediatric (10–16 years) (*n* (%))	30 (7.9)	7 (3.8)	4 (4.9)	-	46 (11.9)	27 (5.81)	1 (2.6)
Adults (*n* (%))	352 (92.1)	177 (96.2)	78 (95.1)	-	342 (89.1)	438 (94.2)	37 (97.4)
**Underlying disease and risk factors (*n* (%))**							
Total number of patients with underlying disease or risk factors	349 ^$^	184	82	27	303	465	38
Diabetes mellitus	187 (53.6) ^a^	120 (65.2)	51 (62.2)	15 (55.6)	172 (56.8)	342 (73.5)	29 (76.3)
Diabetic ketoacidosis	21 (21.6) ^b^	16.9% ^g^	-	-	31 (10.2)	50 (14.6)	3 (7.9)
Solid-organ transplant	9 (2.6) ^a^	-	-	3 (11.1)	19 (6.3)	30 (6.5)	-
HSCT	-	4 (2.2)	-	-	1 (0.3)	6 (1.3)	-
Haematological and solid organ malignancy	16 (4.6) ^a^	14 (7.6)	-	1 (3.7)	23 (7.6)	42 (9)	2 (5.3)
Brach of skin (trauma due to accidents, burns, injection site)	35 (10) ^a^	20 (10.9)	12 (14.6)	6 (22.2)	31 (10.2)	35 (7.5)	8 (21.1)
Pulmonary disease (tuberculosis, COPD, asthma)	1 (0.6) ^c^	-	-	2 (7.4)	21 (6.9)	30 (6.5)	-
Neutropenia	11 (14.6) ^d^	-	-	-	18 (5.9)	12 (2.6)	-
Steroid therapy	28 (8) ^a^	-	-	6 (22.2)	30 (9.9)	17 (3.7)	-
Chronic alcoholism	15 (5.9) ^e^	-	-	-	28 (9.2)	-	-
Chronic kidney disease	24 (32) ^d^	1 (0.5)	1 (1.2)	1 (3.7)	27 (8.9)	93 (20)	2 (5.3)
Human immunodeficiency virus	2 (0.8) ^e^	-	-	-	3 (1)	7 (1.5)	-
Immunocompetent host	45 (12.9) ^a^	10 (5.4)	16 (19.5)	7 (25.9)	32 (10.6)	55 (11.8)	1 (2.6)
^#^ Miscellaneous	53 (31) ^f^	15 (8.2)	8 (9.8)	6 (22.2)	6 (2.0)	143 (30.8)	4 (10.5)

Note: Table values are given in numbers and percentage [*n* (%)]. Abbreviations: ROCM, rhino-orbital-cerebral mucormycosis; HSCT, haematopoietic stem cell transplant; COPD, chronic obstructive pulmonary disease. *Paediatric age in different manuscripts are mentioned in range. ^$^ Data were pooled from three case series [14,15,16]; hence, denominator varies for each underlying illness and risk factor, and denominators are ^a^ *n* = 349; ^b^ *n* = 97; ^c^ *n* = 178; ^d^ *n* = 75; ^e^ *n* = 253; and ^f^ *n* = 171. ^g^ Actual number not mentioned in the study [17]. ^#^ Miscellaneous risk factors include septicaemia, haematological disorders (aplastic anaemia, megaloblastic anaemia, and pancytopenia), autoimmune disease (scleroderma, systemic lupus erythematosus), liver disease (viral hepatitis), immunodeficiency disorders (common variable immunodeficiency), prematurity, bowel perforation, graft-versus-host disease, metabolic acidosis, intensive-care stay, intravenous drug use, iron chelation therapy, high-risk neonate (malnourishment), immunosuppressant drugs, cardiovascular disease, and neurological disease.

**Table 2:** Causative agents of mucormycosis in India.

^ Causative Agents	Chakrabarti et al., 2001; 2006; 2009 [14,15,16]	Manesh et al., 2019 [17]	Chander et. al., 2018 [22]	Prakash et al., 2019 [5]	Patel et al., 2020 [6]	Priya et al., 2020 [18]
Total number of isolated Mucorales	120 ^$^	184	60	**239**	**290**	**25**
*Rhizopus* species	79 (65.8) ^a^	143 (77.7)	28 (46.7)	193 (80.8)	231 (79.7)	14 (56)
*Rhizopus arrhizus*	74 (61.7) ^a^	91 (49.5)	17 (28.3)	124 (51.9)	176 (60.7)	-
*Rhizopus microsporus*	4 (4.2) ^b^	32 (17.4)	9 (15)	30 (12.6)	32 (11)	-
*Rhizopus homothallicus*	1 (3.1) ^c^	-	2 (3.3)	6 (2.5)	22 (7.6)	-
*Apophysomyces* species	31 (25.8) ^a^	20 (10.9)	13 (21.7)	22 (9.2)	23 (7.9)	5 (20)
*Lichtheimia* species	3 (5.3) ^d^	1 (0.5)	8 (13.3)	10 (4.2)	10 (3.5)	1 (4)
*Saksenaea* species	3 (3.4) ^e^	1 (0.5)	5 (8.3)	2 (0.8)	2 (0.7)	-
*Cunninghamella* species	-	1 (0.5)	-	5 (2.1)	3 (1)	-
*Mucor* species	1 (4) ^f^	4 (2.2)	1 (1.7)	3 (1.3)	16 (5.5)	3 (12)
*Rhizomucor* species	2 (2.3) ^e^	1 (0.5)	1 (1.7)	-	4 (1.4)	-
*Syncephalastrum* species	1 (3.1) ^c^	1 (0.5)	4 (6.7)	-	1 (0.4)	-
Nonsporulating Mucorales/other fungi	-	12 (6.5)	-	4 (1.7)	-	2 (8)

Note: Table values are given in numbers and percentage (*n* (%)). ^$^ Data were pooled from three case series [14,15,16]; hence, denominator varies for each species, and denominators are ^a^ *n* = 120; ^b^ *n* = 95; ^c^ *n* = 32; ^d^ *n* = 57; ^e^ *n* = 88; and ^f^ *n* = 25. ^ Current taxonomical names used in the manuscript: *Rhizopus arrhizus* (Syn. *Rhizopus oryzae*), *Rhizopus microsporus* (Syn. *Rhizopus rhizopodoformis*, *Rhizopus azygosporus*) and *Lichtheimia* species (Syn. *Absidia* species) [68]. Species isolated in different manuscripts are: *Rhizopus* (*R. arrhizus*, *R. microsporus*, *R. homothallicus*, *R. asexualis*, and *R. stolonifer*), *Apophysomyces* (*A. elegans*, *A. variabilis*), *Lichtheimia* (*L. corymbifera*, *L. ramosa*), *Saksenaea* (*S. vasiformis*, *S. erythrospora*); *Mucor irregularis*, *Rhizomucor pusillus*; *Syncephalastrum racemosum* and *Cunninghamella bertholletiae*. Few isolates in the different studies are not speciated.

## Data Availability

Not applicable.

## References

[1] Frater J.L., Hall G.S., Procop G.W. (2001). Histologic features of zygomycosis: Emphasis on perineural invasion and fungal morphology. Arch. Pathol. Lab. Med..

[2] Roden M.M., Zaoutis T.E., Buchanan W.L., Knudsen T.A., Sarkisova T.A., Schaufele R.L., Sein M., Sein T., Chiou C.C., Chu J.H. (2005). Epidemiology and outcome of zygomycosis: A review of 929 reported cases. Clin. Infect. Dis..

[3] Jeong W., Keighley C., Wolfe R., Lee W.L., Slavin M.A., Kong D.C.M., Chen S.C.A. (2019). The epidemiology and clinical manifestations of mucormycosis: A systematic review and meta-analysis of case reports. Clin. Microbiol. Infect..

[4] Reid G., Lynch J.P., Fishbein M.C., Clark N.M. (2020). Mucormycosis. Semin. Respir. Crit. Care Med..

[5] Prakash H., Ghosh A.K., Rudramurthy S.M., Singh P., Xess I., Savio J., Pamidimukkala U., Jillwin J., Varma S., Das A. (2019). A prospective multicenter study on mucormycosis in India: Epidemiology, diagnosis, and treatment. Med. Mycol..

[6] Patel A., Kaur H., Xess I., Michael J.S., Savio J., Rudramurthy S., Singh R., Shastri P., Umabala P., Sardana R. (2020). A multi-centre observational study on the epidemiology, risk factors, management and outcomes of mucormycosis in India. Clin. Microbiol. Infect..

[7] Skiada A., Pagano L., Groll A., Zimmerli S., Dupont B., Lagrou K., Lass-Florl C., Bouza E., Klimko N., Gaustad P. (2011). Zygomycosis in Europe: Analysis of 230 cases accrued by the registry of the European Confederation of Medical Mycology (ECMM) Working Group on Zygomycosis between 2005 and 2007. Clin. Microbiol. Infect..

[8] Patel A.K., Patel K.K., Patel K., Gohel S., Chakrabarti A. (2017). Mucormycosis at a tertiary care centre in Gujarat, India. Mycoses.

[9] Kontoyiannis D.P., Yang H., Song J., Kelkar S.S., Yang X., Azie N., Harrington R., Fan A., Lee E., Spalding J.R. (2016). Prevalence, clinical and economic burden of mucormycosis-related hospitalisations in the United States: A retrospective study. BMC Infect. Dis..

[10] Chakrabarti A., Dhaliwal M. (2013). Epidemiology of Mucormycosis in India. Curr. Fungal Infect. Rep..

[11] Chakrabarti A., Singh R. (2014). Mucormycosis in India: Unique features. Mycoses.

[12] Prakash H., Chakrabarti A. (2019). Global Epidemiology of Mucormycosis. J. Fungi.

[13] Pandey M., Singh G., Agarwal R., Dabas Y., Jyotsna V.P., Kumar R., Xess I. (2018). Emerging *Rhizopus microsporus* Infections in India. J. Clin. Microbiol..

[14] Chakrabarti A., Das A., Sharma A., Panda N., Das S., Gupta K.L., Sakhuja V. (2001). Ten Years’ Experience in Zygomycosis at a Tertiary Care Centre in India. J. Infect..

[15] Chakrabarti A., Das A., Mandal J., Shivaprakash M.R., George V.K., Tarai B., Rao P., Panda N., Verma S.C., Sakhuja V. (2006). The rising trend of invasive zygomycosis in patients with uncontrolled diabetes mellitus. Med. Mycol..

[16] Chakrabarti A., Chatterjee S.S., Das A., Panda N., Shivaprakash M.R., Kaur A., Varma S.C., Singhi S., Bhansali A., Sakhuja V. (2009). Invasive zygomycosis in India: Experience in a tertiary care hospital. Postgrad. Med. J..

[17] Manesh A., Rupali P., Sullivan M.O., Mohanraj P., Rupa V., George B., Michael J.S. (2019). Mucormycosis-A clinicoepidemiological review of cases over 10 years. Mycoses.

[18] Priya P., Ganesan V., Rajendran T., Geni V.G. (2020). Mucormycosis in a Tertiary Care Center in South India: A 4-Year Experience. Indian J. Crit. Care Med..

[19] Chakrabarti A., Kaur H., Savio J., Rudramurthy S.M., Patel A., Shastri P., Pamidimukkala U., Karthik R., Bhattacharya S., Kindo A.J. (2019). Epidemiology and clinical outcomes of invasive mould infections in Indian intensive care units (FISF study). J. Crit. Care.

[20] Sindhu D., Jorwal P., Gupta N., Xess I., Singh G., Soneja M., Nischal N., Sethi P., Ray A., Biswas A. (2019). Clinical spectrum and outcome of hospitalised patients with invasive fungal infections: A prospective study from a medical ward/intensive care unit of a teaching hospital in North India. Le Infez. Med..

[21] Chakrabarti A., Sood P., Denning D. Estimating Fungal Infection Burden in India: Mucormycosis Burden as a Case Study. https://www.gaffi.org/wp-content/uploads/P1044.pdf.

[22] Chander J., Kaur M., Singla N., Punia R., Singhal S., Attri A., Alastruey-Izquierdo A., Stchigel A., Cano-Lira J., Guarro J. (2018). Mucormycosis: Battle with the Deadly Enemy over a Five-Year Period in India. J. Fungi.

[23] Bhansali A. (2004). Presentation and outcome of rhino-orbital-cerebral mucormycosis in patients with diabetes. Postgrad. Med. J..

[24] Dayal D., Jain P., Kumar R., Bakshi J., Menon P., Das A., Singhi S., Singh M. (2015). Clinical spectrum and outcome of invasive filamentous fungal infections in children with Type 1 diabetes: North Indian experience. Clin. Pediatr. Endocrinol..

[25] Skiada A., Pavleas I., Drogari-Apiranthitou M. (2020). Epidemiology and Diagnosis of Mucormycosis: An Update. J. Fungi.

[26] Williams R., Colagiuri S., Almutairi R., Montoya P.A., Basit A., Beran D., Besançon S., Bommer C., Borgnakke W., Boyko E. (2019). International Diabetes Federation Diabetes Atlas. Ninth Edition. https://diabetesatlas.org/en/sections/worldwide-toll-of-diabetes.html.

[27] Das A., Oberoi S., Trehan A., Chakrabarti A., Bansal D., Saxena A.K., Sodhi K.S., Kakkar N., Srinivasan R. (2018). Invasive Fungal Disease in Pediatric Acute Leukemia in the Nontransplant Setting: 8 Years’ Experience From a Tertiary Care Center in North India. J. Pediatr. Hematol. Oncol..

[28] Korula A., Abraham A., Abubacker F.N., Viswabandya A., Lakshmi K.M., Abraham O.C., Rupali P., Varghese G.M., Michael J.S., Srivastava A. (2017). Invasive fungal infection following chemotherapy for acute myeloid leukaemia-Experience from a developing country. Mycoses.

[29] Patel M.H., Patel R.D., Vanikar A.V., Kanodia K.V., Suthar K.S., Nigam L.K., Patel H.V., Patel A.H., Kute V.B., Trivedi H.L. (2017). Invasive fungal infections in renal transplant patients: A single center study. Ren. Fail..

[30] Godara S.M., Kute V.B., Goplani K.R., Gumber M.R., Gera D.N., Shah P.R., Vanikar A.V., Trivedi H.L. (2011). Mucormycosis in renal transplant recipients: Predictors and outcome. Saudi J. Kidney Dis. Transpl..

[31] Shekar M., Elumalai R., Elayaperumal I., Yelahanka R.P., Anandkumar D.G., Bandi V.K., Matcha J. (2019). Prevalence and outcome of systemic fungal infections in renal transplant recipients—A tertiary care experience. Saudi J. Kidney Dis. Transpl..

[32] Jayakumar M., Gopalakrishnan N., Vijayakumar R., Rajendran S., Muthusethupathi M.A. (1998). Systemic fungal infections in renal transplant recipients at Chennai, India. Transplant. Proc..

[33] Gupta K. (2001). Fungal infections and the kidney. Indian J. Nephrol..

[34] Gupta K.L., Bagai S., Ramachandran R., Kumar V., Rathi M., Kohli H.S., Sharma A., Chakrabarti A. (2020). Fungal infection in post-renal transplant patient: Single-center experience. Indian J. Pathol. Microbiol..

[35] Almyroudis N.G., Sutton D.A., Linden P., Rinaldi M.G., Fung J., Kusne S. (2006). Zygomycosis in Solid Organ Transplant Recipients in a Tertiary Transplant Center and Review of the Literature. Am. J. Transplant..

[36] Kumar C., Jain P., Wadhwa N., Diwaker P., Khan N. (2017). Nosocomial Jejunal Mucormycosis—An Unusual Cause of Perforation Peritonitis. Iran. J. Pathol..

[37] Garg J., Sujatha S., Garg A., Parija S.C. (2009). Nosocomial cutaneous zygomycosis in a patient with diabetic ketoacidosis. Int. J. Infect. Dis..

[38] Bhadauria D., Etta P., Chelappan A., Gurjar M., Kaul A., Sharma R.K., Gupta A., Prasad N., Marak R.S., Jain M. (2018). Isolated bilateral renal mucormycosis in apparently immunocompetent patients—A case series from India and review of the literature. Clin. Kidney J..

[39] Devana S.K., Gupta V.G., Mavuduru R.S., Bora G.S., Sharma A.P., Parmar K.M., Kumar S., Mete U.K., Singh S.K., Mandal A.K. (2019). Isolated Renal Mucormycosis in Immunocompetent Hosts: Clinical Spectrum and Management Approach. Am. J. Trop. Med. Hyg..

[40] Gupta K.L., Radotra B.D., Sakhuja V., Banerjee A.K., Chugh K.S. (1989). Mucormycosis in patients with renal failure. Ren. Fail..

[41] Kursun E., Turunc T., Demiroglu Y.Z., Alışkan H.E., Arslan A.H. (2015). Evaluation of 28 cases of mucormycosis. Mycoses.

[42] Gupta N., Kumar A., Singh G., Ratnakar G., Vinod K.S., Wig N. (2017). Breakthrough mucormycosis after voriconazole use in a case of invasive fungal rhinosinusitis due to *Curvularia lunata*. Drug Discov. Ther..

[43] Mandhaniya S., Swaroop C., Thulkar S., Vishnubhatla S., Kabra S.K., Xess I., Bakhshi S. (2011). Oral Voriconazole Versus Intravenous Low Dose Amphotericin B for Primary Antifungal Prophylaxis in Pediatric Acute Leukemia Induction. J. Pediatr. Hematol. Oncol..

[44] Kataria S.P., Sharma J., Singh G., Kumar S., Malik S., Kumar V. (2016). Primary breast mucormycosis: FNAC diagnosis of a rare entity. Diagn. Cytopathol..

[45] Hadgaonkar S., Shah K., Bhojraj S., Nene A., Shyam A. (2015). Isolated Mucormycotic Spondylodiscitis of Lumbar Spine-A Rare Case Report. J. Orthop. Case Rep..

[46] Shah K., Nene A. (2017). Spinal mucormycosis. J. Glob. Infect. Dis..

[47] Bharadwaj R. (2017). Sclerosing Mediastinitis Presenting as Complete Heart Block. J. Clin. Diagn. Res..

[48] Krishnappa D., Naganur S., Palanisamy D., Kasinadhuni G. (2019). Cardiac mucormycosis: A case report. Eur. Hear. J. Case Rep..

[49] Bhatt M., Soneja M., Fazal F., Vyas S., Kumar P., Jorwal P., Raj U., Sachdev J., Singh G., Xess I. (2018). Two cases of Osteoarticular Mucor menace: A diagnostic and management conundrum. Drug Discov. Ther..

[50] Urs A., Singh H., Mohanty S., Sharma P. (2016). Fungal osteomyelitis of maxillofacial bones: Rare presentation. J. Oral Maxillofac. Pathol..

[51] Nithyanandam S., Jacob M.S., Battu R.R., Thomas R.K., Correa M.A., D’Souza O. (2003). Rhino-orbito-cerebral mucormycosis. A retrospective analysis of clinical features and treatment outcomes. Indian J. Ophthalmol..

[52] Kolekar J.S. (2015). Rhinocerebral Mucormycosis: A Retrospective Study. Indian J. Otolaryngol. Head Neck Surg..

[53] Bakshi S.S., Das S., Ramesh S., Gopalakrishnan S. (2020). Nasal Mucormycosis: Our experience with 24 cases. Otolaryngol. Pol..

[54] Ramadorai A., Ravi P., Narayanan V. (2018). Rhinocerebral Mucormycosis: A Prospective Analysis of an Effective Treatment Protocol. Ann. Maxillofac. Surg..

[55] Shah K., Dave V., Bradoo R., Shinde C., Prathibha M. (2019). Orbital Exenteration in Rhino-Orbito-Cerebral Mucormycosis: A Prospective Analytical Study with Scoring System. Indian J. Otolaryngol. Head Neck Surg..

[56] Singh V.P., Bansal C., Kaintura M. (2019). Sinonasal Mucormycosis: A to Z. Indian J. Otolaryngol. Head Neck Surg..

[57] Nilesh K., Vande A. (2018). Mucormycosis of maxilla following tooth extraction in immunocompetent patients: Reports and review. J. Clin. Exp. Dent..

[58] Agarwal S., Anand A., Ranjan P., Meena V.P., Ray A., Dutta R., Jadon R.S., Vikram N.K. (2020). Case of mucormycosis of mandible after self-extraction of teeth incidentally detected to have chronic granulomatous disease: Case report and literature review. Med. Mycol. Case Rep..

[59] Lanternier F., Dannaoui E., Morizot G., Elie C., Garcia-Hermoso D., Huerre M., Bitar D., Dromer F., Lortholary O. (2012). French Mycosis Study Group A global analysis of mucormycosis in France: The RetroZygo Study (2005-2007). Clin. Infect. Dis..

[60] Feng J., Sun X. (2018). Characteristics of pulmonary mucormycosis and predictive risk factors for the outcome. Infection.

[61] Kaushik R. (2012). Primary Cutaneous Zygomycosis in India. Indian J. Surg..

[62] Skiada A., Rigopoulos D., Larios G., Petrikkos G., Katsambas A. (2012). Global epidemiology of cutaneous zygomycosis. Clin. Dermatol..

[63] Kaur H., Ghosh A., Rudramurthy S.M., Chakrabarti A. (2018). Gastrointestinal mucormycosis in apparently immunocompetent hosts-A review. Mycoses.

[64] Patra S., Chirla D., Kumar N., Vij M., Samal S. (2012). Unsuspected invasive neonatal gastrointestinal mucormycosis: A clinicopathological study of six cases from a tertiary care hospital. J. Indian Assoc. Pediatr. Surg..

[65] Dioverti M.V., Cawcutt K.A., Abidi M., Sohail M.R., Walker R.C., Osmon D.R. (2015). Gastrointestinal mucormycosis in immunocompromised hosts. Mycoses.

[66] Prakash H., Ghosh A.K., Rudramurthy S.M., Paul R.A., Gupta S., Negi V., Chakrabarti A. (2016). The environmental source of emerging *Apophysomyces variabilis* infection in India. Med. Mycol..

[67] Prakash H., Singh S., Rudramurthy S.M., Singh P., Mehta N., Shaw D., Ghosh A.K. (2020). An aero mycological analysis of Mucormycetes in indoor and outdoor environments of northern India. Med. Mycol..

[68] Walther G., Pawłowska J., Alastruey-Izquierdo A., Wrzosek M., Rodriguez-Tudela J.L., Dolatabadi S., Chakrabarti A., de Hoog G.S. (2013). DNA barcoding in Mucorales: An inventory of biodiversity. Persoonia Mol. Phylogeny Evol. Fungi.

[69] Kokkayil P., Pandey M., Agarwal R., Kale P., Singh G., Xess I. (2017). *Rhizopus homothallicus* Causing Invasive Infections: Series of Three Cases from a Single Centre in North India. Mycopathologia.

[70] Pamidimukkala U., Sudhaharan S., Kancharla A., Vemu L., Challa S., Karanam S.D., Chavali P., Prakash H., Ghosh A.K., Gupta S. (2020). Mucormycosis due to *Apophysomyces* species complex- 25 years’ experience at a tertiary care hospital in southern India. Med. Mycol..

[71] Chakrabarti A., Ghosh A., Prasad G.S., David J.K., Gupta S., Das A., Sakhuja V., Panda N.K., Singh S.K., Das S. (2003). *Apophysomyces elegans*: An emerging zygomycete in India. J. Clin. Microbiol..

[72] Hemashettar B.M., Patil R.N., O’Donnell K., Chaturvedi V., Ren P., Padhye A.A. (2011). Chronic rhinofacial mucormycosis caused by *Mucor irregularis* (*Rhizomucor variabilis*) in India. J. Clin. Microbiol..

[73] Xess I., Mohapatra S., Shivaprakash M.R., Chakrabarti A., Benny G.L., O’Donnell K., Padhye A.A. (2012). Evidence implicating *Thamnostylum lucknowense* as an etiological agent of rhino-orbital mucormycosis. J. Clin. Microbiol..

[74] Chander J., Singla N., Kaur M., Punia R.S., Attri A., Alastruey-Izquierdo A., Cano-Lira J.F., Stchigel A.M., Guarro J. (2017). *Saksenaea erythrospora*, an emerging mucoralean fungus causing severe necrotising skin and soft tissue infections—A study from a tertiary care hospital in north India. Infect. Dis. (Lond. Engl.)..

[75] Cornely O.A., Alastruey-Izquierdo A., Arenz D., Chen S.C.A., Dannaoui E., Hochhegger B., Hoenigl M., Jensen H.E., Lagrou K., Lewis R.E. (2019). Global guideline for the diagnosis and management of mucormycosis: An initiative of the European Confederation of Medical Mycology in cooperation with the Mycoses Study Group Education and Research Consortium. Lancet Infect. Dis..

[76] Jeong W., Keighley C., Wolfe R., Lee W.L., Slavin M.A., Chen S.C.A., Kong D.C.M. (2019). Contemporary management and clinical outcomes of mucormycosis: A systematic review and meta-analysis of case reports. Int. J. Antimicrob. Agents.

[77] Manesh A., John A.O., Mathew B., Varghese L., Rupa V., Zachariah A., Varghese G.M. (2016). Posaconazole: An emerging therapeutic option for invasive rhino-orbito-cerebral mucormycosis. Mycoses.

[78] Marty F.M., Ostrosky-Zeichner L., Cornely O.A., Mullane K.M., Perfect J.R., Thompson G.R., Alangaden G.J., Brown J.M., Fredricks D.N., Heinz W.J. (2016). Isavuconazole treatment for mucormycosis: A single-arm open-label trial and case-control analysis. Lancet Infect. Dis..

